# LISP1 is important for the egress of *Plasmodium berghei* parasites from liver cells

**DOI:** 10.1111/j.1462-5822.2009.01333.x

**Published:** 2009-05-26

**Authors:** Tomoko Ishino, Bertrand Boisson, Yuki Orito, Céline Lacroix, Emmanuel Bischoff, Céline Loussert, Chris Janse, Robert Ménard, Masao Yuda, Patricia Baldacci

**Affiliations:** 1 Institut Pasteur, Biologie et Génétique du Paludisme75724 Paris cedex 15, France; 2 Department of Medical Zoology, Mie University School of MedecineMie 514-0001, Japan; 3 Institut Pasteur, Plateforme Puces à ADN GénopoleParis, France; 4 Institut Pasteur, Plateforme de Microscopie UltrastructuraleParis, France; 5 Department of Parasitology, Leiden University Medical CentreLeiden, the Netherlands

## Abstract

Most Apicomplexa are obligatory intracellular parasites that multiply inside a so-called parasitophorous vacuole (PV) formed upon parasite entry into the host cell. *Plasmodium*, the agent of malaria and the Apicomplexa most deadly to humans, multiplies in both hepatocytes and erythrocytes in the mammalian host. Although much has been learned on how Apicomplexa parasites invade host cells inside a PV, little is known of how they rupture the PV membrane and egress host cells. Here, we characterize a *Plasmodium* protein, called LISP1 (liver-specific protein 1), which is specifically involved in parasite egress from hepatocytes. LISP1 is expressed late during parasite development inside hepatocytes and locates at the PV membrane. Intracellular parasites deficient in LISP1 develop into hepatic merozoites, which display normal infectivity to erythrocytes. However, LISP1-deficient liver-stage parasites do not rupture the membrane of the PV and remain trapped inside hepatocytes. LISP1 is the first *Plasmodium* protein shown by gene targeting to be involved in the lysis of the PV membrane.

## Introduction

*Plasmodium*, the agent of malaria, is an Apicomplexan parasite that cycles through a mosquito and a mammalian host. In the mammalian host, it multiplies by schizogony inside hepatocytes and erythrocytes. The parasite form inoculated by the mosquito, the sporozoite, invades hepatocytes where it transforms into thousands of merozoites, which in turn invade erythrocytes. The successive cycles of merozoite multiplication inside erythrocytes then cause the disease pathology (Miller *et al*., 2002a,b).

The two cell invasive stages of *Plasmodium*, the sporozoite and the merozoite, differentiate intracellularly within a parasitophorous vacuole (PV). The PV membrane (PVM), which is formed upon zoite entry and is mainly derived from the host cell plasma membrane, is devoid of host cell integral proteins and thus prevents PV fusion to endosomal compartments (Lingelbach and Joiner, 1998; Bano *et al*., 2007). The PVM thus provides a safe niche for the maturing parasite and allows passage of vital nutrients and signals via channels and transporters (see review Charpian and Przyborski, 2008). Among the parasite proteins known to be present in the PVM of infected erythrocytes are exported protein 1 (EXP1) (Simmons *et al*., 1987) and several members of the early transcribed membrane proteins (ETRAMP) family, which are integral PVM proteins organized in oligomeric arrays (Spielmann *et al*., 2006). In hepatocytes, the PVM also contains EXP1 (Doolan *et al*., 1996) and the stage-specific ETRAMPs known as UIS3 and UIS4 (Matuschewski *et al*., 2002). The latter two proteins are expressed in sporozoites and early liver stages (LS) and are essential for their growth (Mueller *et al*., 2005a,b; Tarun *et al*., 2007).

Once the parasite has multiplied inside the PV, how the progeny exits the PV and the host cell remains largely unknown (Blackman, 2008). Electron (Aikawa, 1971) and live (Wickham *et al*., 2003) microscopy have provided evidence for two distinct stages in the release of merozoites from erythrocytes, with the successive rupture of the PVM and of the erythrocyte membrane. The two-step model applies to the release of merozoites from hepatocytes (Meis and Verhave, 1988; Sturm *et al*., 2006; 2009). After disruption of the PVM, merozoite-filled membrane-bound extrusions, called merosomes (Sturm *et al*., 2006; 2009; Baer *et al*., 2007; Thiberge *et al*., 2007), detach from the infected hepatocyte before releasing free merozoites into the blood circulation.

Here, we identify and characterize a *Plasmodium* protein, called LISP1 (liver-specific protein 1), which is specifically expressed by the LS and is expressed at high levels late during its development. Inactivation of *Lisp1* in *Plasmodium berghei* indicates that LISP1 is important for the destruction of the PVM surrounding the LS.

## Results

### Identification of *Lisp1*

We previously constructed expressed sequence tag (EST) libraries from various stages of *P. berghei* ANKA parasites, including ookinetes, midgut sporozoites, salivary gland sporozoites, LS isolated from a rat liver 31 h post infection with sporozoites, and merozoites. *Lisp1* was identified as a transcript present only in the LS library and it aligned to four annotated *P. berghei* genes: PB000708.00.0, PB001247.00.0, PB000682.00.0 and PB000250.00.0. An independent *in silico* analysis also selected PB000708.00.0 and PB000682.00.0 as candidate liver-specific genes and real-time PCR analysis confirmed that they were highly expressed in LS. The *Lisp1* transcript is predicted to encode a protein of 3249 amino acids (Accession No. AB231328) with a signal peptide sequence and a potential EF-hand but no other recognizable functional domain (http://www.plasmodb.org). The *Plasmodium yoelii Lisp1* orthologue (PY04499) has recently been detected in a proteomic analysis of infected hepatocytes (Tarun *et al*., 2008). Orthologues of *P. berghei Lisp1* are also found in *Plasmodium falciparum* (PF14_0179) and *Plasmodium vivax* (PVX_085550), but not in other Apicomplexa parasites such as *Cryptosporidium hominis*, *Toxoplasma gondii*, *Theileria annulata* or *Eimeria tenella*.

### *Lisp1* is expressed specifically in LS

**Fig. 1:**
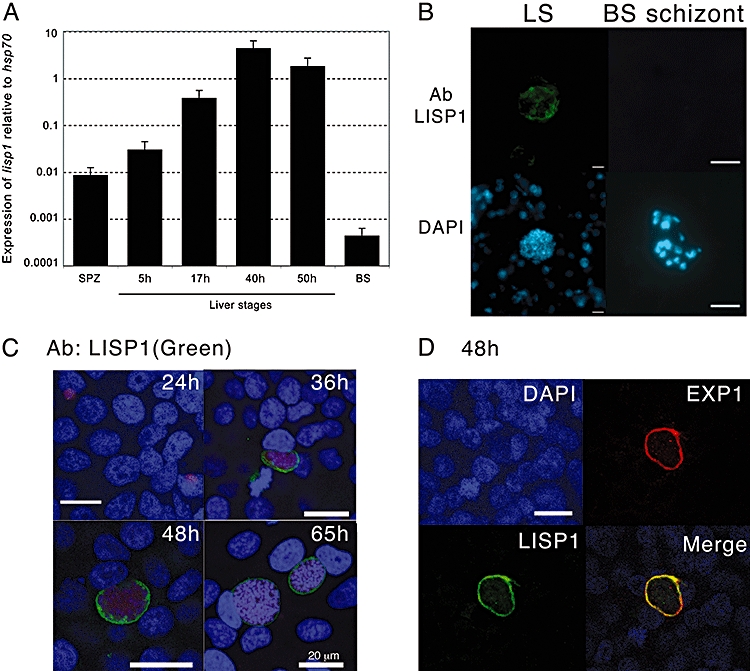
*Pblisp1* is specifically expressed in late liver stages and localizes to the PVM. A. Histogram representation of real-time RT-PCR analysis of *lisp1* relative gene expression in *P. berghei* sporozoites (SPZ), HepG2 cells 5, 17, 40 and 50 h post infection and mixed blood stages (BS). The value was normalized to the expression of *hsp70* mRNA in each sample. Error bars are standard deviation. B. Immunofluorescence analysis of frozen sections of rat liver 48 h post infection (LS) and purified BS schizonts. Samples were incubated with an anti-LISP1 antibody followed by FITC-conjugated secondary antibody and nuclei were stained with DAPI (scale bar 5 μm). C. Micrograph of confocal section of LS. HepG2 cells were fixed at 24, 36, 48 and 65 h post infection with WT ANKA sporozoites, incubated with an anti-LISP1 antibody followed by Alexa 488-conjugated secondary antibody and nuclei stained with DAPI (scale bars 20 μm). D. Micrograph of confocal section of LS. HepG2 cells were fixed 48 h post infection with WT ANKA sporozoites, incubated with anti-LISP1 and anti-EXP1 antibodies followed by Alexa 488-conjugated secondary antibodies and nuclei stained with DAPI (scale bar 20 μm).

To confirm the LS-specific expression of *Lisp1*, real-time PCR analysis was performed on RNA isolated from NK65 blood stages and salivary gland sporozoites, as well as infected HepG2 cells at different time points (Fig. 1A). *Lisp1* messenger RNA (mRNA) was barely detectable in blood stages, present at low levels in sporozoites, and its quantity increased dramatically during parasite development in HepG2 cells with a peak at 40 h (10^4^-fold increase compared with blood stages). Similar results were obtained with RNA isolated from the liver of rats infected with *P. berghei* ANKA, with *Lisp1* expression peaking at 48 h (Fig. S1). Thus *Lisp1* appeared to be most highly expressed at late stages of intrahepatocytic parasite development, when merozoites are formed.

Next, the subcellular localization of LISP1 was addressed by immunofluorescence analysis (IFA) with an anti-LISP1 polyclonal antibody. While no signal was detected in midgut or salivary gland sporozoites (not shown) or purified blood-stage schizonts, parasites developing in the liver of rat 48 h after sporozoite inoculation were brightly stained (Fig. 1B). LISP1 was also detected in infected HepG2 cells at 36, 48 and 65 h post infection (Fig. 1C) where it appeared to be associated with the PVM surrounding the developing LS. To better define the localization of LISP1, IFA was performed with anti-LISP1 and anti-EXP1 antibodies. As shown in Fig. 1D, the two proteins colocalized confirming that LISP1 is present in the PVM and in agreement with the localization of PY04499 in *P. yoelii*-infected hepatocytes (Tarun *et al*., 2008).

### LISP1-deficient parasites have a decreased infectivity to the mammalian host

**Fig. 2:**
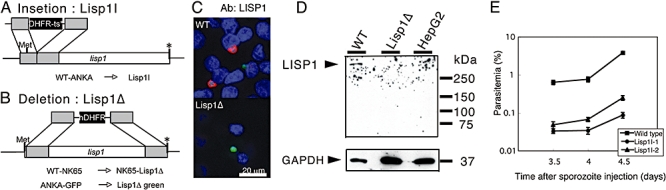
Targeted gene disruption of *lisp1*. A. Schematic representation of *Lisp1I* gene disruption. B. Schema of *Lisp1*Δ gene disruption. Shaded boxes indicate the regions of homology used for double-cross-over recombination; black boxes indicate the selectable marker; Met and the asterisk (*) indicate the initiation and stop codons respectively. C. Absence of LISP1 protein in Lisp1Δ parasites. Microscopy image of HepG2 cells 48 h post infection with WTGreen or Lisp1ΔGreen. LS were labelled with anti-LISP1 antibody and Alexa 647-conjugated secondary antibody (red); nuclei stained with DAPI (blue). D. Western blot analysis of extracts of HepG2 cells 48 h post infection with ANKA (WT) or Lisp1ΔGreen sporozoites. Arrowheads indicate the bands of LISP1 and human GAPDH. E. Lisp1I sporozoites have a decreased infectivity in the mammalian host. A total of 30 000 wild-type or Lisp1I sporozoites, from two independent Lisp1I clones, were injected intravenously into rats and blood parasitaemias were examined by Geimsa staining. The error bars show the standard errors from three rats.

To address the *in vivo* function of LISP1, we inactivated the gene in both wild-type (WT) ANKA and NK65 strains of *P. berghei* by double-cross-over recombination. The endogenous *Lisp1* was either interrupted by the selectable marker (*Lisp1I*) (Fig. 2A) or replaced by the marker (*Lisp1*Δ) (Fig. 2B). The *Lisp1I* modification was introduced in the WT ANKA strain, generating clone Lisp1I. The *Lisp1*Δ modification was introduced in WT NK65, generating the clone NK65Lisp1Δ, and in an ANKA strain expressing green fluorescent protein (GFP) (Janse *et al*., 2006a), generating clone Lisp1ΔGreen. Southern blot analysis confirmed the expected structure of all recombinant loci (Fig. S2). The absence of LISP1 protein in Lisp1ΔGreen parasites was confirmed by IFA of infected HepG2 cells with an anti-LISP1 antibody (Fig. 2C). Using the same antibody, Western blot analysis of extracts from HepG2 cells 48 h post infection with WT ANKA sporozoites detected the expected 380 kDa band, whereas no band was observed upon infection with Lisp1Δ parasites (Fig. 2D).

All the *Lisp1* mutant parasite clones displayed similar growth rates in mouse blood stages and similar infectivity in mosquitoes, and yielded similar numbers of salivary gland sporozoites when compared with WT parasites (not shown). This indicated that *Lisp1* has no essential role in erythrocytic and mosquito stages. The infectivity of mutant sporozoites was measured by checking the emergence of blood-stage parasites in rats injected intravenously with Lisp1I salivary gland sporozoites. The parasitaemias obtained with mutant sporozoites were significantly different (*P*= 1.4 × 10^−7^) from those obtained with WT sporozoites (Fig. 2E) and indicated a 15-fold decrease in infectivity. Similar results were obtained with all *Lisp1* mutants (Fig. S3), so the two ANKA-derived mutants, Lisp1I and Lisp1ΔGreen, were used for further analysis.

### Mutant LS parasites are impaired in merozoite release

**Fig. 3:**
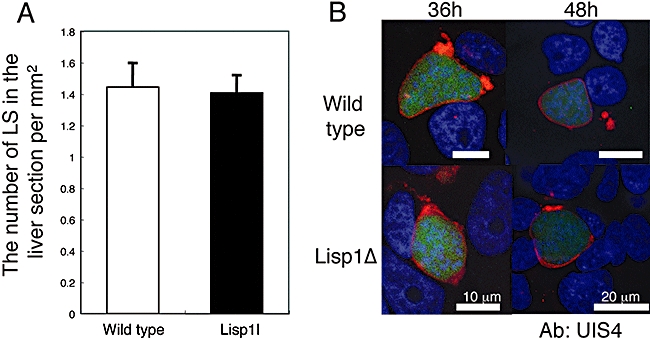
*Lisp1*-defective parasites develop normally, within a PVM, into late-stage LS. A. Histogram representation of the number of LS *in vivo*. A total of 300 000 wild-type or Lisp1I sporozoites were injected intravenously into rats and 48 h later the number of WT (white box) and Lisp1I (black box) LS were counted in the liver sections. Bars indicate the standard deviation from three rats. B. Confocal micrograph of PVM in *Lisp1*-defective parasites. HepG2 cells 36 and 48 h post infection with WT (Wild type) or Lisp1ΔGreen sporozoites. LS parasites (green) labelled with an anti-UIS4 antibody (red) and nuclei stained with DAPI (blue).

We then examined the intrahepatocytic development of mutant parasites. First, 300 000 ANKA or Lisp1I sporozoites were injected intravenously into rats and 48 h later the number of LS were counted in liver sections immuno-stained with anti-CS antibodies. No significant difference was observed between the numbers of ANKA and Lisp1I LS parasites (Fig. 3A). Second, the development of WT and Lisp1ΔGreen LS was assessed with an anti-UIS4 antibody. The presence of a PVM was detected until 48 h in both WT and mutant parasites (Fig. 3B). Furthermore, when WT and Lisp1I (or Lisp1ΔGreen) sporozoites were incubated with HepG2 cells there was no significant difference in the number of LS parasites at 48 or 64 h (data not shown). Finally, we assessed by real-time imaging the development of WT and mutant LS at 24, 36 and 48 h post infection of HepG2 cells. Again there was no significant difference in the size of WT compared with mutant LS over this period (Fig. S4).

To examine hepatic merozoite development, the numbers of merozoites released from HepG2 cells 65 h post infection were compared between the WT and mutant parasites. To internally control experiments, mixed infections were performed with a 1:1 ratio of sporozoites of Lisp1ΔGreen and of WTRed, a WT ANKA derivative that expresses red fluorescent protein (RFP) from the *eef1α* promoter (Sturm *et al*., 2009) and the ratio of green versus red merozoites (G/R) released in the culture supernatant was calculated (see Table 1). To verify that green and red merozoites were detected with similar efficiency, cells were co-infected with a 1:1 ratio of WTGreen and WTRed sporozoites. An average G/R merozoite ratio of 1 was found, confirming that green- and red-fluorescent merozoites were detected with similar efficiency. In contrast, co-infections with Lisp1ΔGreen and WTRed parasites gave an average G/R merozoite ratio of 0.1:1. The 10-fold reduction in the number of merozoites released by mutant LS suggested that LISP1 might be important for merozoite formation within, or escape from infected hepatocytes.

**Table 1:** The ratio of merozoites released from HepG2 cells co-infected with either WTGreen and WTRed or Lisp1ΔGreen and WTRed sporozoites.

Experiment No.	WTGreen : WTRed	Lisp1ΔGreen : WTRed
1	1.1:1	0.20:1
2	1.0:1	0.10:1
3	1.1:1	0.24:1
4		0.12:1
5		0.059:1
6	0.8:1	0.12:1
7		0.11:1
Ave	1.0:1	0.11:1

The average (Ave) ratio from seven independent experiments is shown.

**Fig. 4:**
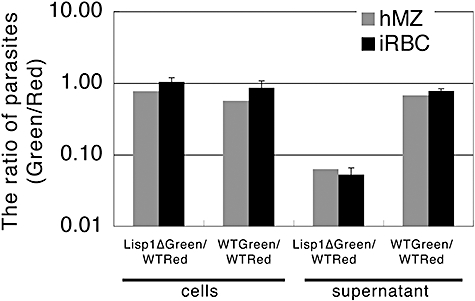
*Lisp1* mutant LS parasites are defective in the release of merozoites. Graph showing the ratio of Green : Red hepatic merozoites (hMZ) collected at 65 h from adherent HepG2 cells (left) or the supernatant (right) co-infected with Lisp1ΔGreen and WTRed or WTGreen and WTRed sporozoites and the ratios found in blood stages (iRBC) after injection of these merozoites into mice. The results obtained from the supernatant and adherent cells are indicated. The average and standard deviation from four mice are shown.

To evaluate the number of hepatic merozoites formed in *lisp1* mutant LS, adherent HepG2 cells, co-infected with Lisp1ΔGreen and WTRed sporozoites, were recovered after 65 h and mechanically disrupted to release the merozoites. The ratio of Lisp1ΔGreen to WTRed merozoites was ∼1 (Fig. 4, left panel cells, hMZ), indicating that mutant parasites generated normal numbers of merozoites. Next, the infectivity of mutant merozoites was tested. When the approximately 1:1 ratio of Lisp1ΔGreen to WTRed merozoites collected from the disrupted HepG2 cells was injected into mice, the same ratio was found in the ensuing blood stages (Fig. 4, left panel cells, iRBC). Further, when supernatants from the mixed infections collected at 65 h were injected intravenously into mice, the ratio of Lisp1ΔGreen : WTRed blood-stage parasites (Fig. 4, right, supernatant) was the same as the ratio of injected hepatic merozoites and was maintained after several multiplication cycles, showing that the merozoites released from *lisp1* mutant LS were indeed infectious (see also Fig. S5) and had similar infectivity to red blood cells compared with WT.

Taken together, these data indicate that in the absence of LISP1 infectious merozoites are formed normally but are not released efficiently from the LS.

### Mutant LS do not rupture the PVM

**Fig. 5:**
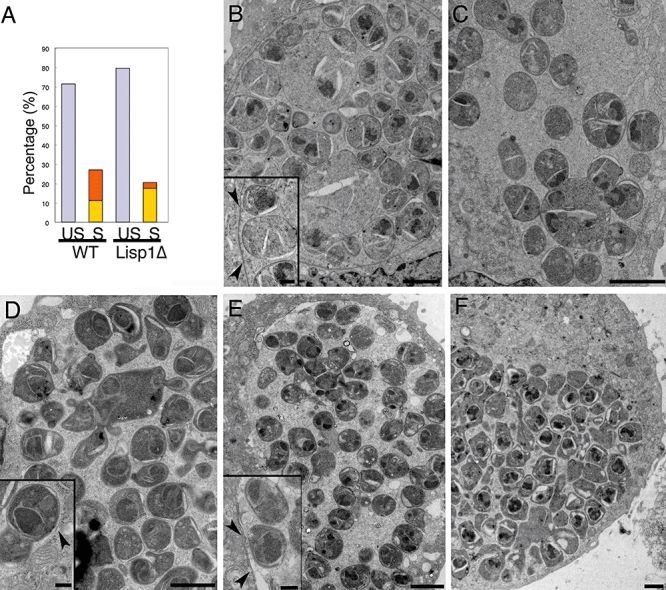
*Lisp1* mutant merozoites remain within a PVM. A. Histogram of the percentage of 58 h WTGreen and Lisp1ΔGreen unsegmented LS with no merozoites (US, lilac), and segmented LS containing merozoites (S) with (yellow) or without (orange) a visible PVM. B–F. Representative examples of hepatic schizonts 58 h (B–D) and 61 h (E and F) post infection. (B) WTGreen with PVM; (C) WTGreen without PVM; (D) Lisp1Δ LS with PVM; (E and F) Lisp1Δ LS at 61 h containing many merozoites within a membrane boundary; scale bars are 2 μm. Insets show mature merozoites; scale bar is 500 nm and arrow heads indicate membrane.

We next examined the morphology of late stages of LS development by transmission electron microscopy (TEM). HepG2 cells were infected with WTGreen or Lisp1ΔGreen sporozoites, and 58 or 61 h later the infected cells were isolated by fluorescence-activated cell sorting and processed for TEM. As shown in Fig. 5A, there was no difference between the development of WT and *Lisp1* mutant parasites at 58 h. Indeed, the proportion of LS containing morphologically mature merozoites was very similar (20–30%). However, about 50% of the WT parasites were no longer surrounded by a visible PVM (Fig. 5B and C) whereas the majority of *Lisp1* mutant parasites were packed within a membrane (Fig. 5D). At 61 h, we were unable to observe WT LS containing merozoites presumably because the breakdown of the PVM rendered them too fragile to survive the sorting procedure. On the contrary, mutant-infected cells were found and the majority of these contained numerous merozoites inside a visible PVM (Fig. 5E and F). The ultrastructure of these merozoites appeared normal as assessed by the presence of a nucleus, rhoptries and a membrane coat (see Fig. S6). These observations strongly support the role of LISP1 in the PVM breakdown and merozoite egress from the host cell.

### SERA1 localization is modified in LISP1-defective LS

**Fig. 6:**
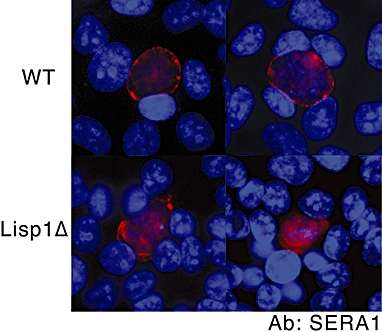
Localization of SERA1 is modified in *Lisp1* mutant LS parasites. HepG2 cells were infected with wild type (WT, top) or Lisp1ΔGreen sporozoites (Lisp1Δ, bottom) and 48 h later the LS were stained an anti-SERA1 antibody (red) and nuclei stained with DAPI (blue).

Several lines of evidence strongly suggest that SERA cysteine proteases play an important role in the rupture of the PVM (Sturm *et al*., 2009) so we assessed the distribution of SERA-1 protein in LISP1-defective LS. HepG2 cells were infected with WTGreen or Lisp1ΔGreen sporozoites and 48 h later the LS were stained with anti-SERA1 antibody. In WT LS the distribution of SERA gave a punctuated signal located at the PVM whereas in the mutant LS, the signal was more diffuse and possibly extended beyond the PVM (Fig. 6 and Fig. S7). These data show that in the absence of LISP1 the distribution of SERA1 is modified.

## Discussion

Egress from the host cell is a fundamental and recurrent step of the *Plasmodium* life cycle. Evidence has now accumulated indicating that the process occurs in two distinct steps, with the rupture of the PVM preceding the rupture of the host cell membrane. The molecular bases of these processes, however, remain unknown.

Earlier work performed with erythrocytic forms of the parasite (reviewed in Blackman, 2008) has suggested that the breakdown of the PV and erythrocyte membranes both depend on proteases but have distinct bases, since treatment with E64, a cysteine protease inhibitor, selectively blocks PVM rupture. Several lines of evidence suggest that the cysteine proteases of the serine repeat antigen (SERA) family (Debrabant *et al*., 1992), which share a central papain-like domain, play a major role in these processes. This gene family contains nine members in *P. falciparum* and five in rodent-infecting species (Arisue *et al*., 2007). In *P. falciparum*, SERA proteins are expressed late during the schizogonic cycle (Hodder *et al*., 2003; Le Roch *et al*., 2003; 2004) and accumulate in the PV lumen (Knapp *et al*., 1989; 1991), and the paralogues encoding SERA5 and SERA6 cannot be inactivated (Miller *et al*., 2002a,b; McCoubrie *et al*., 2007). In *P. berghei*, four of the five SERA-encoding genes are upregulated late during LS maturation, and SERA2 and SERA3 are released in the hepatocyte cytoplasm during merozoite formation (Schmidt-Christensen *et al*., 2008). Cysteine protease activity is known to be important for the destruction of the LS-enclosing PVM, as well as in the subsequent liberation of merosomes, since the two processes are E64-sensitive (Sturm *et al*., 2006; Sturm and Heussler, 2007). The available data thus indicate that the SERA cysteine proteases play an important role in the disruption of the PVM, although some additional, non-cysteine protease activity is probably required for disrupting the host cell membrane. Gene targeting in *P. berghei* also showed that SERA8, called egress cysteine protease 1 (ECP1), is important for sporozoite egress from extracellular oocysts in the gut of the mosquito vector, inside which parasites extensively multiply (Aly and Matuschewski, 2005).

We have characterized here a novel protein, LISP1, which is produced by late LS and associates with their enclosing PVM. Inactivation of *Lisp1* in *P. berghei* decreased 15-fold the LS capacity to generate a blood infection in mice. *In vitro*, LISP1-deficient hepatic merozoites were normally formed inside HepG2 cells, and displayed normal infectivity to erythrocytes. This indicates that LISP1 does not play an important role in the function of the PVM, either as a structural protein or as a transporter. However, the PVM of defective LS was not degraded and the merozoites remained trapped inside hepatocytes. The timing of LISP1 production, its localization and the phenotype of the defective mutant thus suggest that LISP1 plays an important role in the rupture of the LS-enclosing PVM. Nevertheless, since LISP1-deficient parasites give rise to a delayed blood infection in mice, release of some merozoites can occur.

The molecular function of LISP1, annotated as a hypothetical protein with a predicted signal peptide but no recognizable functional domain, remains unknown. Being located at the PVM, LISP1 might act as a membrane receptor of the proteases and/or might be involved in their activation/processing at the membrane. In erythrocytic merozoites, processing of the SERA proteases depends on the subtilisin-like serine protease SUB1, which is abundant in schizont stages and is discharged from organelles named exonemes into the PV just prior to egress (Yeoh *et al*., 2007). LISP1 might thus participate in the further processing of SERA proteases at the PVM, activating their membrane-destabilizing capacity. In the absence of LISP1, the SERA proteins might still be secreted through the PVM without being activated, hence the observed phenotype. Alternatively, LISP1 might be a target of the proteases, mediating membrane dislocation itself upon hydrolysis or processing. Finally, since LISP1 was not detected at the hepatocyte membrane, it seems unlikely that it is also directly involved in merosome formation and hepatocyte/merosome membrane disruption.

Most members of the Apicomplexa phylum, to which *Plasmodium* belongs, invade host cells and multiply within the confines of a PVM that they must eventually breach (Blackman, 2008). In spite of this conserved behaviour, it is striking that all *Plasmodium* products identified so far as being involved in the function/structure of the PVM, such as the ETRAMPs, or in its disruption, such as the SERA proteases, are specific to the genus. Moreover, these products come in stage-specific paralogues adapted to the particular host cell harbouring the PV. LISP1, which appears to be specific to the *Plasmodium* genus, is important for parasite egress from hepatocytes but dispensable for egress from erythrocytes. LISP1 thus adds to the notion of exquisitely specific mechanisms involved in the lysis of the PVM by Apicomplexa parasites.

## Experimental procedures

### *P. berghei* strains and the infection of mice, rats and mosquitoes

ANKAGreen is ANKA 507m6cL1 which expresses GFP from the *eef1α* promoter (Janse *et al*., 2006a) and was obtained from MR4 (MRA-867), deposited by C.J. Janse and A.P. Waters; ANKARed (ANKA L733), expressing RFP from *eef1α* promoter, is described in Sturm *et al*. (2009). Infection of mice, *Anopheles stephensi* mosquitoes and isolation of salivary gland sporozoites were performed as previously described (Thiberge *et al*., 2007). All studies on animals followed the guidelines on the ethical use of animals from the European Communities Council Directive of 24 November 1986 (86/609/EEC).

### Real-time reverse transcription polymerase chain reaction (RT-PCR)

Two independent total RNA preparations were made from mixed infected blood stages, salivary gland sporozoites at day 21 post infection and HepG2 cells 5, 17, 40 and 50 h post infection. PCR conditions were one cycle at 95°C for 10 min, 40 cycles at 95°C for 15 s, 55°C for 15 s and 60°C for 45 s. *Lisp1* gene expression was normalized by the *hsp70* (PB001074.01.0) gene expression in each sample. Analysis was performed using the ΔCt method (User Bulletin 2, Applied Biosystems) using the blood stages as reference.

Primers used: *lisp1*F 5′-GCCAAATGCTAAACCTAATG-3′; *lisp1*R 5′-TGGGTTTGTATTGTATGCAC-3′; *hsp70*F 5′-TGCAGCAGATAATCAAACTC-3′; *hsp70*R: 5′-ACTTCAATTTGTGGAACACC-3′.

### Rabbit anti-LISP1 antibody preparation

LISP1 amino acids 1392–1437 were fused to glutathione S-transferase (GST) using the pGEX 6P-1 system (Amersham Bioscience, Uppsala, Sweden). The protein was purified on a GST column and used to immunize rabbits. Specific antibodies were affinity purified on an *N*-hydroxysuccinimide-activated column (Amersham Bioscience) coupled with recombinant protein. The antibody titre was 1.07 mg ml^−1^.

### Immunofluorescence analysis

Purified parasites were fixed in acetone for 2 min and the samples incubated with rabbit anti-LISP1 antibodies then FITC-conjugated secondary antibody (Zymed, South San Francisco, CA, USA). For nuclear staining, 0.02 μg ml^−1^ 4′, 6-diamidino-2-phenylindole (DAPI) was added to the secondary antibody solution.

For IFA of LS in infected HepG2 cells, fixation was either 4% paraformaldehyde (PFA) + 0.1% triton or acetone, and incubation was with anti-LISP1 antibodies and Alexa 647-conjugated secondary antibody. PFA (4%) fixation and 0.1% triton permeabilization were used with the anti-UIS4 antibody. Methanol fixation was used with anti-EXPI and anti-SERA1 antibodies.

### Targeted disruption of *lisp1* and genotypic analysis

#### 

##### Construction of the *lisp1I* targeting vector

Two fragments of 853 and 801 bp were amplified from genomic DNA with primers: 5′-GGGGAGCTCGTCTATTTTTGATACGATATGTGCACATGC-3′ and 5′-GGGGGATCCCTTGAAGGCGATTAACTATATTGTCGC-3′; 5′-GGGCTCGAGCGAATCAGTGTCGCTTGATATTTTG-3′ and 5′-GGGGGTACCCTGGGTTTGTATTGTATGCACCTAAGG-3′ and cloned either side of the selectable marker gene in pBluescript (Stratagene, La Jolla, CA, USA). For Southern analysis ANKA genomic DNA was digested with SphI and hybridized with a probe made from amplification with primers: 5′-GGGGAGCTCGTCTATTTTTGATACGATATGTGCACATGC-3′ and 5′-GGGGGATCCCTTGAAGGCGATTAACTATATTGTCGC-3′.

##### Construction of the *Lisp1Δ* targeting vector

Two fragments of 630 and 600 bp were amplified from genomic DNA with primers 5′-CGATGCGGGCCCGAGAATACAACTTACTAGGAAATGCAC-3′ and 5′-AGCTGGCGCGCCCTTGCATCTTCAGACATGTTATTTTCGA-3′; 5′-CCAGTGAGTGCGGCCGCGGCTAAAGCTCGATGTCGTATTCAAGAA-3′ and 5′-AGCTGGCGCGCCCTGGGAATTTTTCAATTTCTTCCATTTG-3′. These primers were tailed with restriction sites for ApaI, SmaI, NotI and AscI respectively. The fragments were cloned in the vector pBC-hDHFR vector (B. Boisson, unpublished). This vector is pBC SK (+) (Stratagene), modified to introduce an AscI restriction site, and contains a human dihydrofolate reductase (hDHFR) selection cassette with the 5′ untranslated region of the *eef1α* gene and the 3′ untranslated region of the DHFR-TS gene cloned between SmaI and NotI of pBC SK (+). Transfection and selection of recombinant parasites was performed as previously described (Janse *et al*., 2006b). Southern analysis was performed on SphI-restricted genomic DNA hybridized with the probe indicated on Fig. S2.

## Evaluation of sporozoite infectivity *in vivo*

Sporozoites collected from mosquito salivary glands were injected intravenously into 3-week-old female Wistar rats (Japan SLC, Hamamatsu, Japan) (*n* = 5) or 4-week-old C57BL/6 mice (Janvier, France). Parasitaemias were checked at indicated time points by Geimsa-stained blood smear.

## LS development assay *in vivo*

A total of 300 000 Lisp1I or WT sporozoites were injected into 3-week-old Wistar rats and 48 h later, the livers were perfused with phosphate-buffered saline (PBS) then 4% PFA. After inclusion in 20% sucrose, 20 μm frozen sections were prepared on a Leica cryostat. The sections were stained with anti-CS antiserum and the number of LS in six sections from each rat was counted. A total of 1619 Lisp1I, 1877 WT LS were counted.

## LS development in HepG2 cells

HepG2 cells were grown in DMEM + Glutamax-1 media (Gibco) supplemented with 10% FCS (PAA laboratories GmbH) at 37°C in the presence of 5% CO_2_. The day before infection 4–5.0 × 10^4^ were plated per well in eight-well chamber slides (Nalge Nunc International, Rochester, NY, USA) and 24 h later WT and/or mutant sporozoites (5.0 × 10^4^) were added. The medium was changed daily. The LS were detected by immunofluorescence staining as described above.

## Real-time imaging of LS development in HepG2 cells

HepG2 cells were co-infected with WTRed and Lisp1ΔGreen sporozoites. The development of 27 WT and 28 mutant LS was followed from 24 to 48 h post infection by acquisition of images at 60 min intervals with a PerkinElmer spinning disc confocal microscope. The area was calculated using ImageJ software.

## Detection of LISP1 by immunoblot

HepG2 cells were infected with WTGreen or Lisp1ΔGreen sporozoites as described above. After 48 h the cells from each well were lysed in Novex® Tris-Glycine sodium dodecyl sulfate (SDS) sample buffer (Invitrogen) and immediately stored at −80°C. Prior to loading on a 4–15% gradient acrylamide gel (Bio-Rad), β mercaptoethanol was added (0.7 M final) and the samples were heated for 5 min at 95°C. Molecular weight markers were Precision Plus Protein (Bio-Rad). After electrophoresis the proteins were transferred to a Hybond-ECL nitrocellulose membrane (Amersham Biosciences). The membrane was first incubated with a 1:2000 dilution of the anti-LISP1 antibody then with a 1:10 000 dilution of a chicken anti-rabbit IgG horseradish peroxidase (HRP)-conjugated antibody (Santa Cruz). The presence of antibodies was revealed with the SuperSignal® West Femto kit (Thermo Scientific). The membrane was then incubated with a 1:10 000 dilution of HRP-conjugated rabbit polyclonal anti-GAPDH (glyceraldehyde-3-phosphate dehydrogenase) (Santa Cruz) and the same kit used to reveal the presence of antibodies. The signals were scanned using Quantity One software (Bio-Rad) and quantified with NanoDrop ND-1000 software (Thermo Fischer Scientifique).

## Estimation of number of hepatic merozoites

HepG2 cells were co-infected with equal numbers of either WTRed and WTGreen or WTRed and Lisp1ΔGreen sporozoites. Sixty-five hours later the supernatants were taken, centrifuged for 3 min at 12 000 r.p.m. and the pellet re-suspended in PBS. Cells were scraped from the wells and mechanically disrupted by passing through a Omnican® 30G insulin needle. The samples were placed on Ibidi slides and observed with a Zeiss Axio observer Z1 microscope.

## TEM analysis of infected HepG2 cells

WTGreen or Lisp1ΔGreen sporozoites were added to HepG2 cells and 58 or 61 h later cells were collected, fixed with 0.4% PFA and GFP-positive cells enriched by sorting on a FACSAria (BD Biosciences). Sorted cells were fixed with 2.5% glutaraldehyde in 0.1 M cacodylate buffer at 4°C for 24 h. Then, cell pellets were embedded in agarose type IV (Sigma, Chemical, Saint Louis, USA). After several washes in 0.1 M cacodylate buffer, samples were post-fixed for 1 h with 1% osmium tetroxide (Merck, Darmstadt, Germany) in the same buffer. After dehydration in a graded ethanol series, the samples were embedded in Epon resin and polymerized. Contrasted ultra-thin sections (60 nm) were observed in a JEM 1010 Transmission Elecron Microscope (Jeol, Tokyo, Japan). A total of 103 Lisp1ΔGreen and 62 WT LS were observed at 58 h.

## Statistical analysis

The data presented in Figs 1A, 2E and 3A were analysed by a Kruskal–Wallis rank sum test. The data presented in Fig. 4 were analysed by a Mann–Whitney test.

## Data deposition

Sequences from the EST libraries were deposited in the DDBJ website 2007. The LISP1 sequence reported in this article has been deposited in the GenBank database (Accession No. AB231328).

## Supporting information

**Fig. S1.** Histogram representation of real-time RT-PCR analysis of *lisp1* relative gene expression in rat livers 24, 48 and 60 h post infection. Total liver RNA was isolated 24, 48 and 60 h post infection with RNAgents Total RNA Isolation System (Promega, Madison, WI, USA) and cDNAs made. Lisp1 was amplified with primers 5′-GCCCCCTGGCGATCTTAATTT-3′ and 5′-TAAACG TTTCAGGGGGCGAT-3′. The expression was normalized to rat glyceraldehyde-3-phosphate dehydrogenase, amplified with primers 5′-TGATTCTACCCACGGCAAGTT-3′ and 5′-TGATGGG TTTCCCATTGATGA-3′. Error bars are standard deviation.

**Fig. S2.** Gene inactivation of *lisp1*.

A. Double-crossing-over strategy used to generate Lisp1I mutant parasites. Genomic DNA of ANKA (WT) and two clones of Lisp1I digested with SphI and hybridized with a 5′ probe indicated by a thick black line.

B. Double-crossing-over strategy used to generate Lisp1Δ clones in NK65 and WTGreen. Genomic DNA was digested with SphI and hybridized with a 5′ probe indicated by a thick black line.

**Fig. S3.** Infectivity of Lisp1ΔGreen sporozoites compared with WTGreen (wild type). A total of 25 000 salivary gland sporozoites were injected intravenously into C57Bl/6 mice and blood-stage parasitaemias checked daily. Bars indicate standard errors.

**Fig. S4.** The size of *lisp1*-defective LS is normal.

A. Histogram representation of the size of LS in HepG2 cells co-infected with WTRed (Wild type, white box) and Lisp1ΔGreen (black box) sporozoites. The LS parasites were detected by their fluorescence and imaged by spinning disk confocal microscopy and the relative area calculated with ImageJ software.

B. Sample of sorted HepG2 cells 58 h post infection with WTGreen (top) or Lisp1ΔGreen sporozoites used for TEM.

**Fig. S5.** Lisp1I merozoites express AMA1. Merozoites collected from the supernatant of HepG2 cells infected with ANKA and Lisp1I sporozoites were incubated with an anti-AMA1 antibody and Alexa 546-conjugated secondary antibody.

**Fig. S6.** Transmission electron micrographs of WT and mutant LS 59 h post infection.

A. WT LS with numerous merozoites in the host cytoplasm and absence of PVM. The aspect of the nucleus indicates that the cell is dying.

B. Lisp1ΔGreen LS with many merozoites and a visible PVM. C and D. Detail of Lisp1ΔGreen hepatic merozoites. The merozoites appear normal at the ultrastructural level with the presence of a nucleus, rhoptry and coated membrane.

**Fig. S7.** Immunofluorescence analysis of LS 48 h post infection of HepG2 cells with WT sporozoites. Samples were incubated with an anti-LISP1 antibody and an anti-SERA1 antibody followed by Alexa-conjugated secondary antibodies. LISP1 labelling in green, left; SERA1 labelling in red, centre; merged image, right.
