# Supplementary figures and images for: LISP1 is important for the egress of Plasmodium berghei parasites from liver cells
Source: Cell Microbiol. 2009 May 26;11(9):1329–39. doi: 10.1111/j.1462-5822.2009.01333.x (PMC2774474; doi:10.1111/j.1462-5822.2009.01333.x)

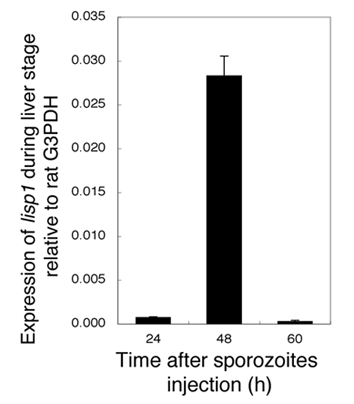

Supplement: Supplementary file 1 [file cmi0011-1329-SD1.tif]

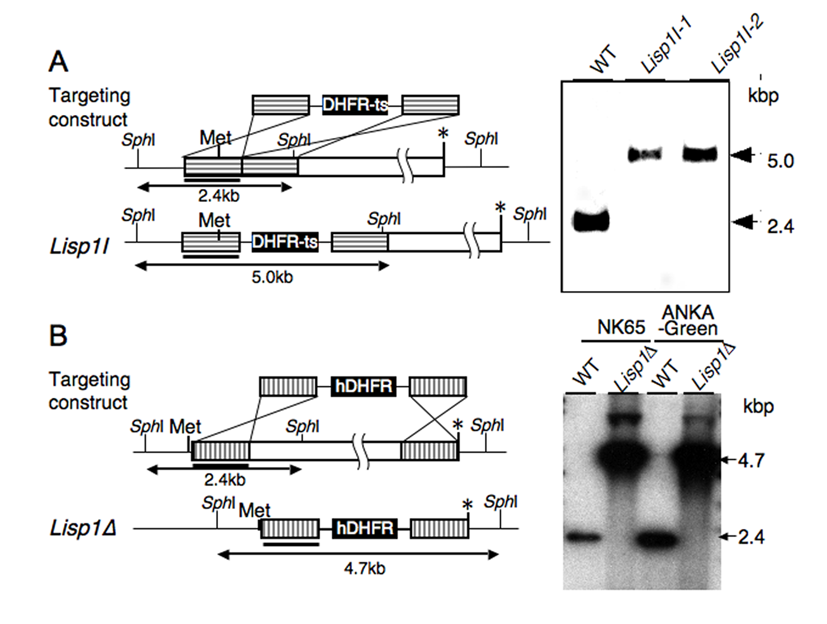

Supplement: Supplementary file 2 [file cmi0011-1329-SD2.tif]

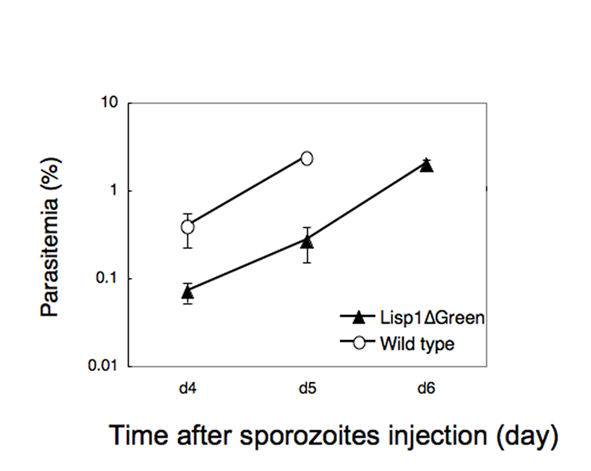

Supplement: Supplementary file 3 [file cmi0011-1329-SD3.tif]

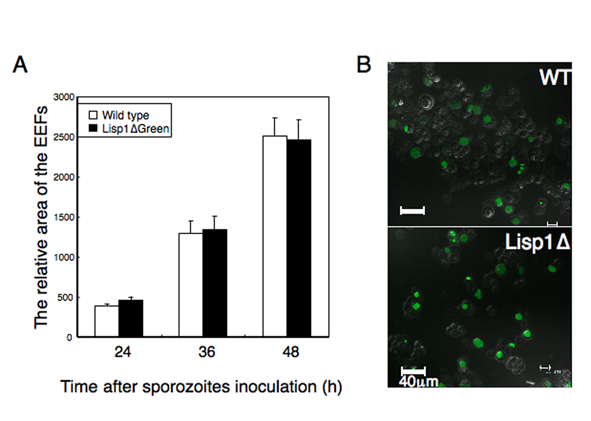

Supplement: Supplementary file 4 [file cmi0011-1329-SD4.tif]

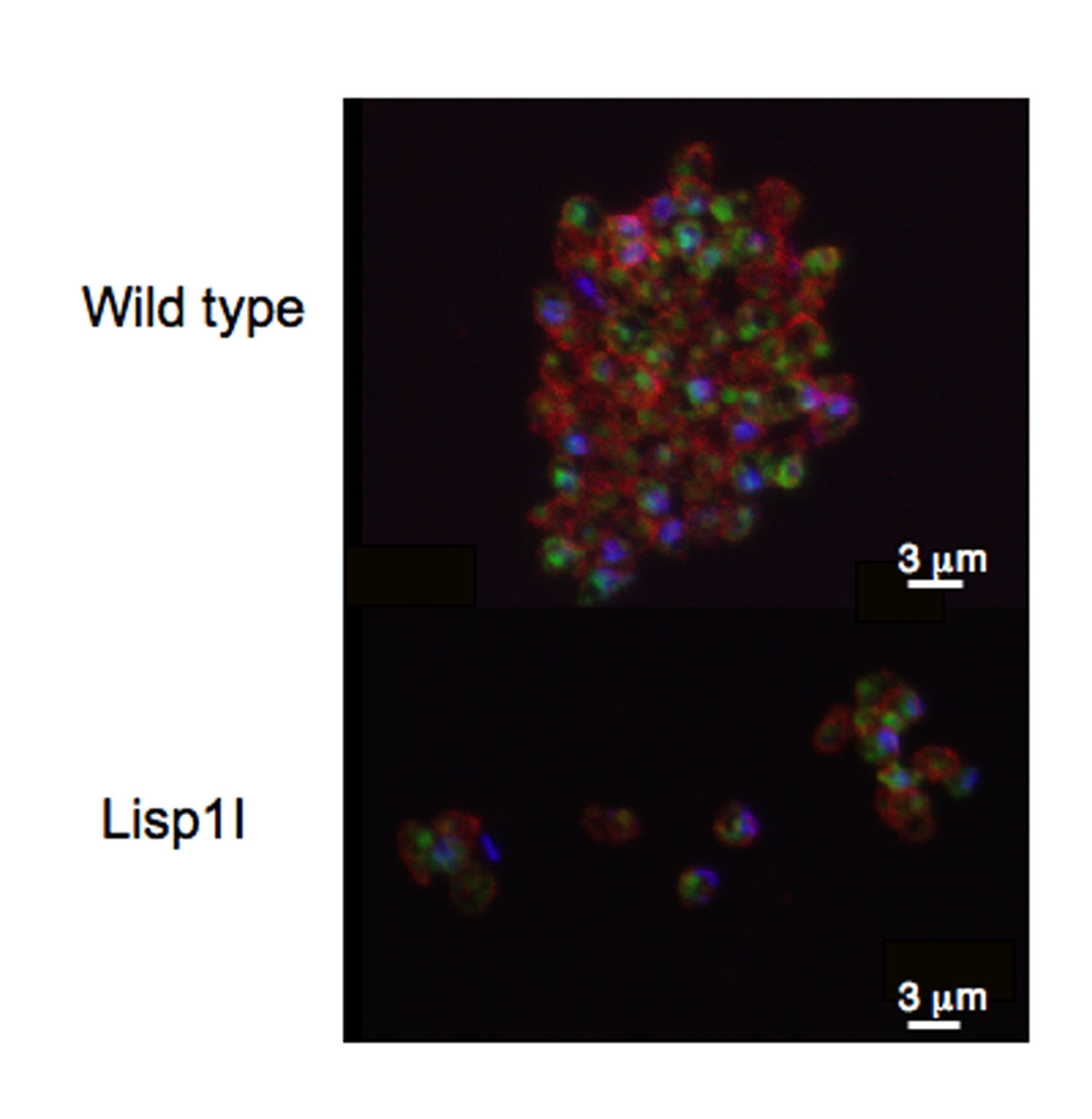

Supplement: Supplementary file 5 [file cmi0011-1329-SD5.jpg]

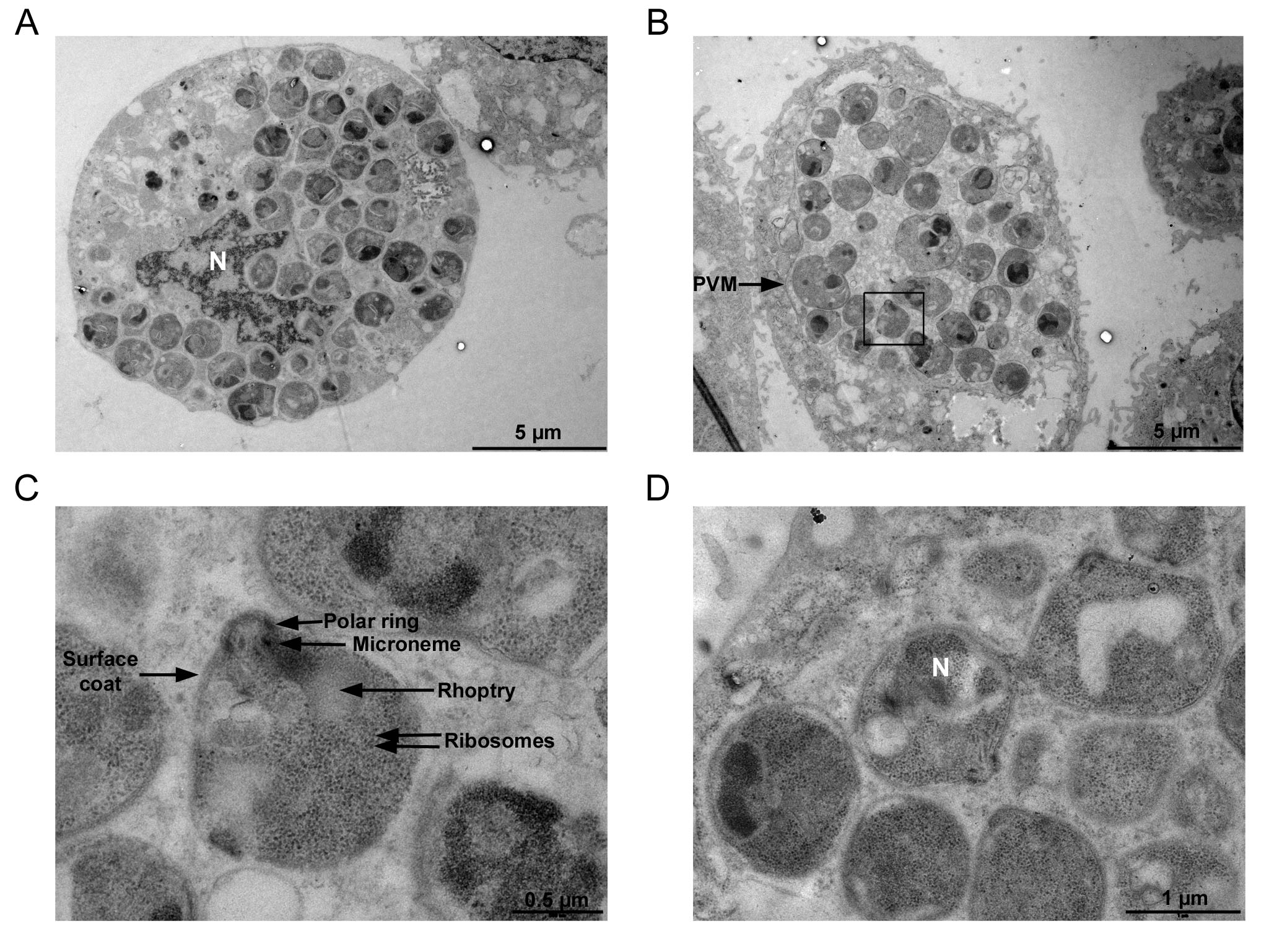

Supplement: Supplementary file 6 [file cmi0011-1329-SD6.jpg]

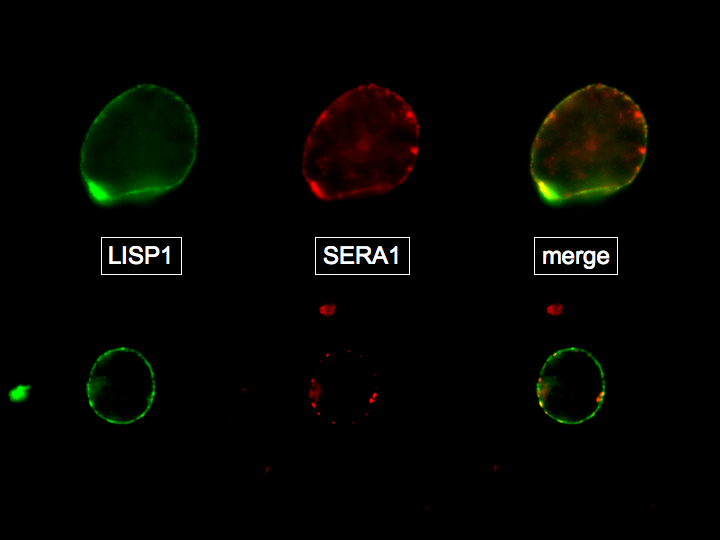

Supplement: Supplementary file 7 [file cmi0011-1329-SD7.tif]
